# Demonstration of entry tear and disrupted intima in asymptomatic chronic thrombosed type B dissection with non-obstructive angioscopy

**DOI:** 10.1136/bcr-2018-225268

**Published:** 2018-04-24

**Authors:** Satoru Takahashi, Sei Komatsu, Mitsuhiko Takewa, Kazuhisa Kodama

**Affiliations:** Cardiovascular Center, Osaka Gyoumeikan Hospital, Osaka, Japan

**Keywords:** cardiovascular medicine, clinical diagnostic tests

## Description

**Figure 1:**
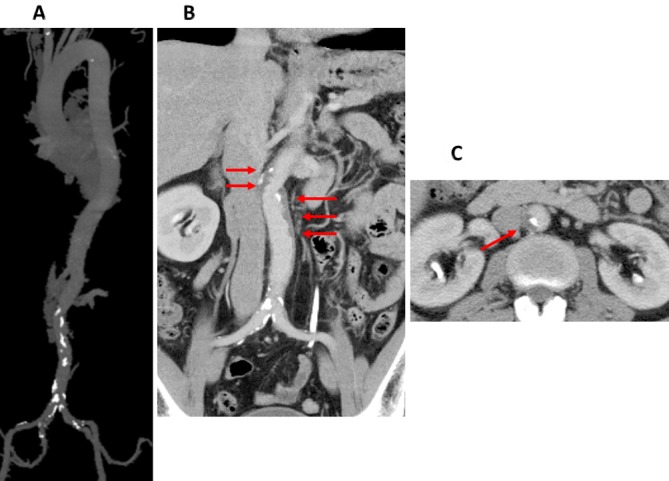
CT angiography images of the aorta. (A) Maximum intensity projection. Calcification deposited in the infrarenal abdominal aorta; however, a few calcifications were found in other sections. (B) Coronal view of the abdominal aorta. Red arrows show the false lumen. (C) Axial image at the T12–L1 level. Red arrows show the false lumen with calcification.

A 49-year-old man was referred to our hospital for atypical chest pain, without severe abdominal or back pain. He had a history of smoking and dyslipidaemia. His ECG showed no ST-T elevation. Coronary CT angiography (CTA) suggested moderate left anterior descending artery stenosis. CTA screening for aortic atherosclerosis showed significant calcification (figure 1A) and a crescent-shaped, mural low-density area in the infrarenal abdominal aorta (figure 1B,C). This was thought to be an intramural haematoma or a thrombosed false lumen. Calcified spots were deposited at the boundaries between the lumen and the low-density area (figure 1B,C). An intramural haematoma or a thrombosed type B dissection can be asymptomatic. Invasive coronary angiography showed no significant stenosis.

**Figure 2:**
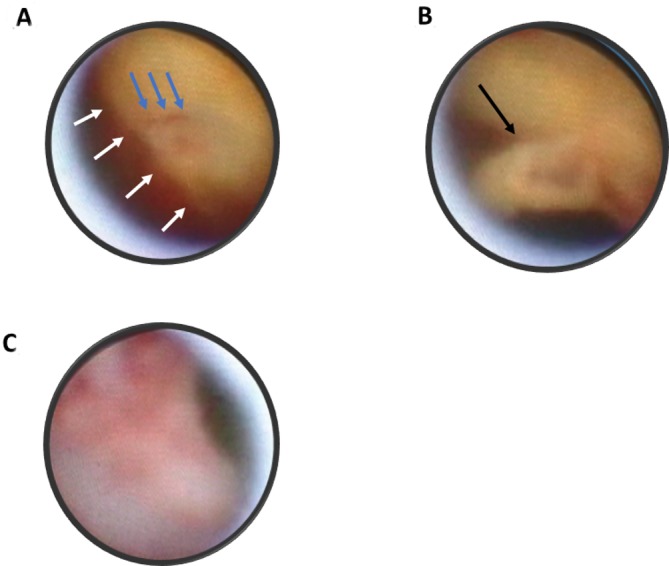
Angioscopic images of aortic ruptured plaques on the surface of the type B thrombosed dissection. (A) Clear boundary (white arrows) of the bright yellow area with smooth surface and red area with irregular surface and bleeding from the entry (blue arrows). (B) Mobile white thrombus or flap (black arrow). (C) Salmon pink-coloured intima.

Non-obstructive angioscopy (NOA) with a left brachial approach was performed to evaluate the aortic atherosclerosis.^1^ Aortic plaques were observed from the right common iliac artery to the aortic arch. NOA initially detected the clear boundary of the dissection. The intima of one side was reddish compared with that of the normal side. Bleeding was then detected from an approximately 1 mm entry tear close to the boundary (figure 2A, video 1), and a mobile white thrombus or flap was detected (figure 2B, video2). The salmon pink-coloured intima with its rough surface was consistently observed (figure 2C). With detection of the entry tear, asymptomatic thrombosed type B dissection was diagnosed.

**Video 1:** Angioscopic video of the boundary of the aortic dissection and normal aortic wall and bleeding from the entry.

**Video 2:** Angioscopic video of the mobile white thrombus or flap.

Blood eosinophil levels on the following day and 30 days later were 0/μL and 63/μL, respectively, with a baseline level of 83/μL. Serum creatinine levels on the following day and 30 days later were 0.82 mg/dL and 0.79 mg/dL, respectively, with a baseline level of 0.86 mg/dL. C reactive protein levels on the following day and 30 days later were 0.79 mg/dL and 0.43 mg/dL, respectively, with a baseline level of 0.59 mg/dL. These results suggested that no embolic complication occurred after NOA. No embolic complications were observed in 324 consecutive patients within 24 hours after aortic NOA.^2^

The patient’s course has been uneventful with conservative management for 2 years. CTA is performed every 6 months, as aortic size was normal. However, there is a limitation to the size-based determination of indications for treatment to prevent unexpected rupture of an aortic aneurysm with dissection,^1^ because asymptomatic aortic plaque ruptures may be associated with aortic fragility.^3^ CTA may overlook such injuries because of limited spatial resolution.^3^ CT and NOA are capable of spatial resolution at 500 µm and 150 µm, respectively. A disadvantage of CTA may be that images are static and tissue characterisation is only based on CT values, and thus may be unable to differentiate non-calcified plaques. The advantage of NOA in the diagnosis of aortic dissection is its ability to detect the entry tear and intimal injury with direct observation and to record these with both images and videos.

NOA may be useful in diagnosing aortic dissection and disrupted intima, including the boundaries of the dissection and entry tear, mobile thrombus and flaps.

**Learning points**

- Asymptomatic thrombosed type B dissection may be safely diagnosed with non-obstructive angioscopy (NOA).
- NOA is superior to CT angiography because of its higher spatial resolution and its capability of direct recording with both still images and videos.
- The size-based determination of indications for treatment to prevent unexpected rupture of an aortic aneurysm and dissection is a limitation.

## References

[1] Komatsu S, Ohara T, Takahashi S, et al Early detection of vulnerable atherosclerotic plaque for risk reduction of acute aortic rupture and thromboemboli and atheroemboli using non-obstructive angioscopy. Circ J 2015;79:742–50. doi:10.1253/circj.CJ-15-0126 25766407 10.1253/circj.CJ-15-0126

[2] Komatsu S, Yutani C, Ohara T, et al Spontaneous ruptured aortic plaques are more frequent and smaller than thought: Novel insights from angioscopy. JACC. In Press.

[3] Komatsu S, Ohara T, Takahashi S, et al Improving the visual field in coronary artery by with non-obstructive angioscopy: dual infusion method. Int J Cardiovasc Imaging 2017;33:789–96. doi:10.1007/s10554-017-1079-1 28176183 10.1007/s10554-017-1079-1

